# Stage II/III rectal cancer with intermediate response to preoperative radiochemotherapy: Do we have indications for individual risk stratification?

**DOI:** 10.1186/1477-7819-8-27

**Published:** 2010-04-13

**Authors:** Thilo Sprenger, Hilka Rothe, Klaus Jung, Hans Christiansen, Lena C Conradi, B Michael Ghadimi, Heinz Becker, Torsten Liersch

**Affiliations:** 1 Department of General and Visceral Surgery, University Medical Center Göttingen, Georg-August-University, Göttingen, Germany; 2 Department of Pathology, University Medical Center Göttingen, Georg-August-University, Göttingen, Germany; 3 Department of Medical Statistics, University Medical CenterGöttingen, Georg-August-University, Göttingen, Germany; 4 Department of Radiotherapy and Radiooncology, University Medical Center Göttingen, Georg-August-University, Göttingen, Germany

## Abstract

**Background:**

Response to preoperative radiochemotherapy (RCT) in patients with locally advanced rectal cancer is very heterogeneous. Pathologic complete response (pCR) is accompanied by a favorable outcome. However, most patients show incomplete response. The aim of this investigation was to find indications for risk stratification in the group of intermediate responders to RCT.

**Methods:**

From a prospective database of 496 patients with rectal adenocarcinoma, 107 patients with stage II/III cancers and intermediate response to preoperative 5-FU based RCT (ypT2/3 and TRG 2/3), treated within the German Rectal Cancer Trials were studied. Surgical treatment comprised curative (R0) total mesorectal excision (TME) in all cases. In 95 patients available for statistical analyses, residual transmural infiltration of the mesorectal compartment, nodal involvement and histolologic tumor grading were investigated for their prognostic impact on disease-free (DFS) and overall survival (OS).

**Results:**

Residual tumor transgression into the mesorectal compartment (ypT3) did not influence DFS and OS rates (p = 0.619, p = 0.602, respectively). Nodal involvement after preoperative RCT (ypN1/2) turned out to be a valid prognostic factor with decreased DFS and OS (p = 0.0463, p = 0.0236, respectively). Persistent tumor infiltration of the mesorectum (ypT3) and histologic tumor grading of residual tumor cell clusters were strongly correlated with lymph node metastases after neoadjuvant treatment (p < 0.001).

**Conclusions:**

Advanced transmural tumor invasion after RCT does not affect prognosis when curative (R0) resection is achievable. Residual nodal status is the most important predictor of individual outcome in intermediate responders to preoperative RCT. Furthermore, ypT stage and tumor grading turn out to be additional auxiliary factors. Future clinical trials for risk-adapted adjuvant therapy should be based on a synopsis of clinicopathologic parameters.

## Background

Multimodal treatment strategies and optimized surgical procedures with total mesorectal excision (TME) led to a significant improvement in rectal cancer therapy within the last 15 years [1-5]. Nevertheless, a postulation of more individualized approaches in rectal cancer treatment exists for some time. To some extent this postulation is realized in stage dependant therapy as preoperative RCT is recommended only in locally advanced (stage II/III) rectal cancer [6,7].

After preoperative RCT, therapy-induced downsizing effects have widely been described as important prognostic factors [8,9]. Local response to neoadjuvant long-term RCT is very heterogeneous and varies between no morphologic alteration and complete shrinkage with pathologic complete response (pCR). Anyway, in most patients a moderate local response with variable residual tumor infiltration depth (ypT2/3) results [10]. This group of patients with intermediate response is of particular interest as it represents the largest subcategory, which prognostically is difficult to classify. Within this group, tumor transgression of the actual rectal wall and infiltration of the mesorectal compartment (≥ ypT3) constitutes a distinction with unknown impact on prognosis. Subclassification of pT3 rectal cancers has already turned out to be a reliable risk factor for cancer recurrence in patients undergoing primary surgery [11-13] but its prognostic relevance after preoperative RCT is still unclear.

According to TNM classification [14], tumor invasion depth of the mesorectal compartment is divided into subgroups depending on the precise infiltration depth: *(y)pT3a to (y)pT3d*. Therefore (y)pT3 category spans the invasion of only a few tumor cells beyond the muscularis propria to a complete infiltration of the mesorectum, nearby reaching the visceral peritoneum or contiguous organs [14].

A risk-adapted stratification of patients after preoperative RCT and TME-based surgery is crucial for adjuvant treatment strategies in individual patients. Currently, a beneficial impact of adjuvant chemotherapy (CT) is discussed controversely [15,16]. To date, standardized application of adjuvant CT is guaranteed only within randomized clinical trials and clinicopathologic indications for risk stratification in patients after multimodal therapy are extensively missing.

In this study we investigated 107 patients with intermediate local response to preoperative 5-FU based RCT (ypT2/3) and curative (R0) surgery. The aim of this investigation was to clarify the impact of residual tumor infiltration of the mesorectal compartment (≥ ypT3b), nodal status (ypN) and histologic tumor grading on DFS and OS and to evaluate their relevance within an individual risk stratification model in intermediate responders to RCT.

## Methods

### Eligibility

This study included patients with locally advanced rectal cancer (stage II/III) and moderate RCT-induced histopathologic tumor regression (TRG 2 and 3 according to the Dworak classification[17]) and concomitant residual ypT2/3 status. All tumors were located not more than 16 cm from the anal verge, measured by rigid rectoscopy.

Patients with clinical evidence of distant metastatic disease were excluded from the actual investigation and received individual multimodal treatment.

### Clinical Assessments

Pretherapeutical staging procedures consisted of rigid rectoscopy, flexible colonoscopy, endorectal ultrasound (ErUS), magnetic resonance imaging (MRI) of the pelvis and computed tomography (CT) scans of chest, liver and pelvis. Staging results were conferred and interdisciplinary discussed before initiation of multimodal treatment. Clinical tumor stages (cT, cN, cUICC) were determined by ErUS, pelvic MRI, and CT scans.

### Multimodal Treatment

Preoperative treatment included fractional radiation with cumulative 50.4 Gy (28 × 1.8 Gy) in 3- or 4-field technique. Concomitant chemotherapy consisted of either 5-Fluorouracil (5-FU) monotherapy in 84 patients or a combined 5-FU + Oxaliplatin regime in 23 patients. Six weeks after completion of neoadjuvant treatment all patients underwent standardized TME-based surgery. Subsequently, postoperative systemic therapy was applied according to the preoperative treatment regimen (5-FU monotherapy or combined 5-FU + Oxaliplatin) and the actual study protocol.

### Pathologic Assessment

Quality assessment of the surgical specimens was performed according to the MERCURY criteria[18] and was followed by standardized pathological diagnostics of the specimens by an experienced gastrointestinal pathologist. The complete tumor area and all detectable mesorectal lymph nodes were paraffin-embedded and investigated using hematoxylin and eosin staining.

### Pathological Staging/Grading

Pathological staging included ypTNM stage according to the current TME classification[14], tumor differentiation grading, evaluation of proximal, distal and circumferential resection margins and intra- and extramural vascular and perineural invasion. Nodal staging included histological evaluation of all detected lymph nodes and statement of lymph node ratio in all cases with regard to the consensual minimum number of 12 nodes per specimen [14,19]. RCT-induced tumor regression was denoted on the basis of a semi-quantitative 5 point grading system according to established methods [10,17]. Subdivision of ypT3 status was performed in accordance to subdivision of pT3 status [20] and is shown in Table 1.

**Table 1:** Subdivision of yp T3 status

ypT3a	Residual Tumor Infiltration into the Mesorectum **< 1 mm**
ypT3b	Residual Tumor Infiltration into the Mesorectum **> 1 - 5 mm**

ypT3c	Residual Tumor Infiltration into the Mesorectum **> 5 - 15 mm**

ypT3d	Residual Tumor Manifestation into the Mesorectum **> 15 mm**

**Figure 1:**
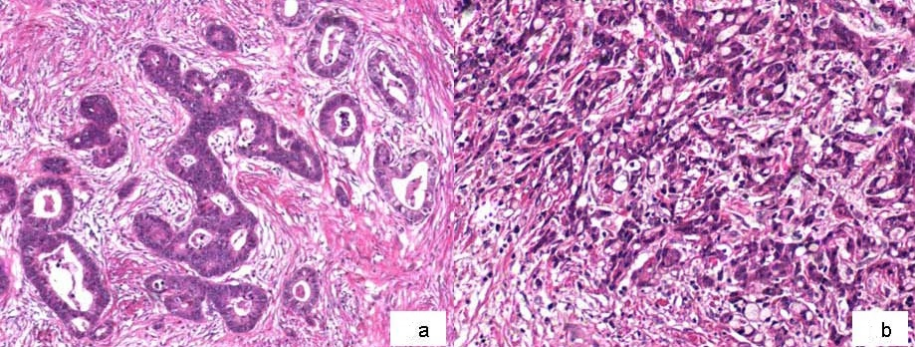
**Histopathologic differentiation: well/moderate differentiated residual tumor cell clusters after RCT with preserved glandular growth pattern (High Grade Tumors; Figure 1a)**. Poorly differentiated residual tumor cells with non-glandular formations (Low Grade Tumors; Figure 1b).

Histopathologic differentiation of *residual tumor cells *was evaluated after preoperative RCT and subdivided into two categories (Figure 1):

High Grade Differentiation: well and moderate differentiated residual tumor cell clusters with preserved glandular growth pattern

Low Grade Differentiation: less and poorly/undifferentiated residual tumor cell clusters with non-glandular growth pattern

### Follow-up

Follow-up assessments included measurement of blood parameters including serum carcinoembryonic antigen and abdominal ultrasound every 3 months. Rectoscopy and CT scans were performed every 6 months within the first 3 years and every 12 months thereafter. Local recurrence was defined as cancer relapse within the pelvic region or the site of the anastomosis. Distant metastatic disease appeared as any tumor manifestation outside the pelvis.

### Statistical Methods

DFS and OS probabilities were estimated by the Kaplan-Meier method and compared between the different levels of clinicopathologic parameters (ypT, ypN, cN and tumor differentiation grading) by a Cox proportional hazards regression model. The ypT and ypN parameters were additionally evaluated in a multivariate analysis.

The distributions of ypN status within the two subgroups of ypT (ypT2/3a and ypT3b-d) were compared by Fisher's exact test. The number of detected lymph nodes between nodal positive and negative patients was compared with the Mann-Whitney-U test. The significance level was set to α = 5% for all tests. All analyses were performed with the free software R (version 2.6,http://www.r-project.org).

## Results

### Patient Population

**Figure 2:**
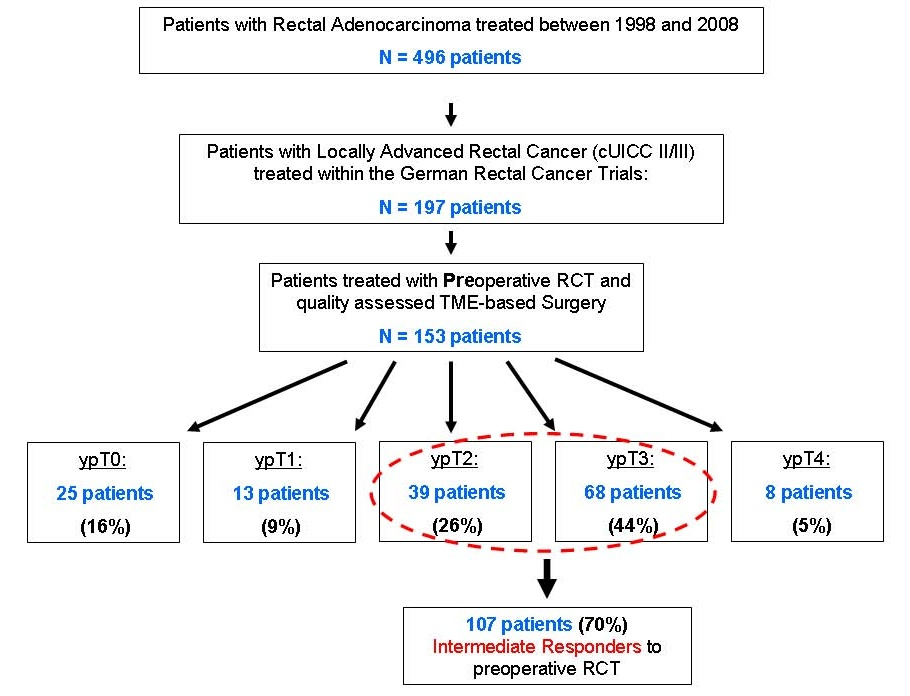
**Distribution of ypT stage in 153 patients treated with preoperative RCT within clinical phase II/III trials**. 107 patients (70%) manifested as intermediate responders with irradiation-induced tumor regression (TRG 2/3)[17] and ypT2 and ypT3 category.

Between January 1998 and June 2008 496 patients with histologically confirmed adenocarcinoma of the rectum were treated at our department. Of these, 153 patients with locally advanced (stage II/III) rectal cancer received preoperative RCT within the German rectal cancer trials (CAO/ARO/AIO-94 [6], XelOx [21] and the ongoing CAO/ARO/AIO-04 trial) and underwent quality assessed curative (R0) TME-based surgery. The approval from the medical ethics committee of the University of Göttingen and informed consent from all subjects were obtained prior to enrolment into the respective study. Following TME, 107 patients (70%) were defined as intermediate responders to neoadjuvant RCT. Of these, 95 were included in the present analysis (Figure 2).

At the time of surgery, 3 patients without previous evidence of distant metastatic disease presented with synchronous liver metastases (stage IV), as detected by manual liver palpation and intraoperative ultrasound. These patients likewise had evidence of residual mesorectal lymph node metastases within the surgical specimen and were excluded from survival analysis.

During a median follow-up period of 42 months (range: 4 - 126 month), 9 of the 107 patients died of non-cancer-related disease and were excluded from cancer specific survival analyses. Seventeen (15.9%) patients had cancer relapse, with 15 cases of separate distant metastatic disease and 2 cases of local recurrence combined with synchronous distant metastases. No isolated local recurrence occurred. Three patients failed statistical analyses due to occult synchronous hepatic metastases detected during surgery (ypUICC IV). In summary, 95 patients were included into survival analysis.

Patient characteristics, pretherapeuthical staging results and treatment procedures of all 107 intermediate responders to preoperative RCT are presented in Table 2. The postsurgical and pathological staging results are summarized in Table 3.

**Table 2:** Clinical findings and treatment procedures

Feature	Number of Patients (n = 107)	%*
Gender		
Male	83	78
Female	24	22

Age (years)		
Median	62.3	
Range	36 - 81	

Tumor Distance from Anal Verge (cm)		
0-6	53	50
>6-12	47	44
>12-16	7	7

cT Stage		
1	0	0
2	1	1
3	98	92
4	8	7

cN Stage		
Positive	82	77
Negative	25	23

cUICC Stage		
I	0	0
II	25	23
III	82	77
IV	0	0

Neoadjuvant Treatment		
50.4 Gy + standard 5-FU	84	79
50.4 Gy + intensified 5-FU/Oxaliplatin	23	21

Surgical Procedure (including TME)		
Low Anterior Resection (incl. laparoscopic)	63 (2)	59 (2)
Abdominoperineal Resection (incl. laparoscopic)	43 (1)	40 (1)
Hartmann's Procedure	1	1

**Table 3:** Pathological findings

Feature	Number of Patients (n = 107)	%
ypT Stage		
2	39	36
3a	16	15
3b	30	28
3c	20	19
3d	2	2

ypN Stage		
0	69	64
1	28	26
2	10	9

ypUICC Stage		
2	69	64
3	35	33
4 *	3	3

Resection Status		
R0	107	100
R1	0	0

Tumor Regression Grading^17^		
Grade 2	59	55
Grade 3	48	45

Circumferential Resection Margin		
Negative	107	100
Positive	0	0

Histologic Differentiation Grading after RCT		
High Grade Differentiation	73	68
Low Grade Differentiation	34	32

Nodal Yield (nodes)		
0-12	5	5
12-18	30	28
18-30	50	47
>30	22	21

Cancer Recurrence		
Total	17	16
Local	0	0
Local + Distant	2	2
Distant	15	14

* Patients were not included in statistical analysis

### Pathological Staging Results

When comparing pretherapeuthical clinical staging with pathological staging results, RCT-induced T-Level downsizing was achieved in 42% of patients (n = 45). Eight tumors, initially staged as cT4 were downsized to ypT2 (n = 2), ypT3b (n = 2), ypT3c (n = 3) and ypT3d (n = 1). Thirty-seven tumors, previously staged as cT3 were downsized to ypT2 status. Nodal downstaging from cUICC III to ypUICC II stage was achieved in 41% of patients (n = 44).

The median number of detected and histopathologically evaluated lymph nodes per specimen was 21 (range: 6 - 79). In 68% of specimens, lymph node yield accounted for ≥ 18 nodes. Fewer than 12 nodes, which is the consensual number according to TNM criteria, were found in a total of 5 specimens (4.7%).

In patients with extended lymph node recovery, lymph node metastases were detected more frequently. However, this finding was not statistically significant (p = 0.06). In detail, the median number of lymph nodes found in the ypN0 group was 19 (range: 6 - 79) compared to 24 (range: 7 - 77) in the ypN1/2 group.

Of 95 patients included in cancer specific survival analyses, 63 (66.3%) were classified as node negative (ypN0), and 32 (33.7%) patients presented with residual lymph node metastases (ypN1/2) after RCT. Fifty patients (54.3%) had intramural tumor infiltration with a maximal infiltration of ≤ 1 mm beyond the muscularis propria (ypT2/3a). Forty-two patients (45.7%) had advanced ypT status with distinct (>1 mm) tumor invasion into the mesorectal compartment (ypT3b-d).

### Pathologic Staging Parameters: Correlation with Survival

**Figure 3:**
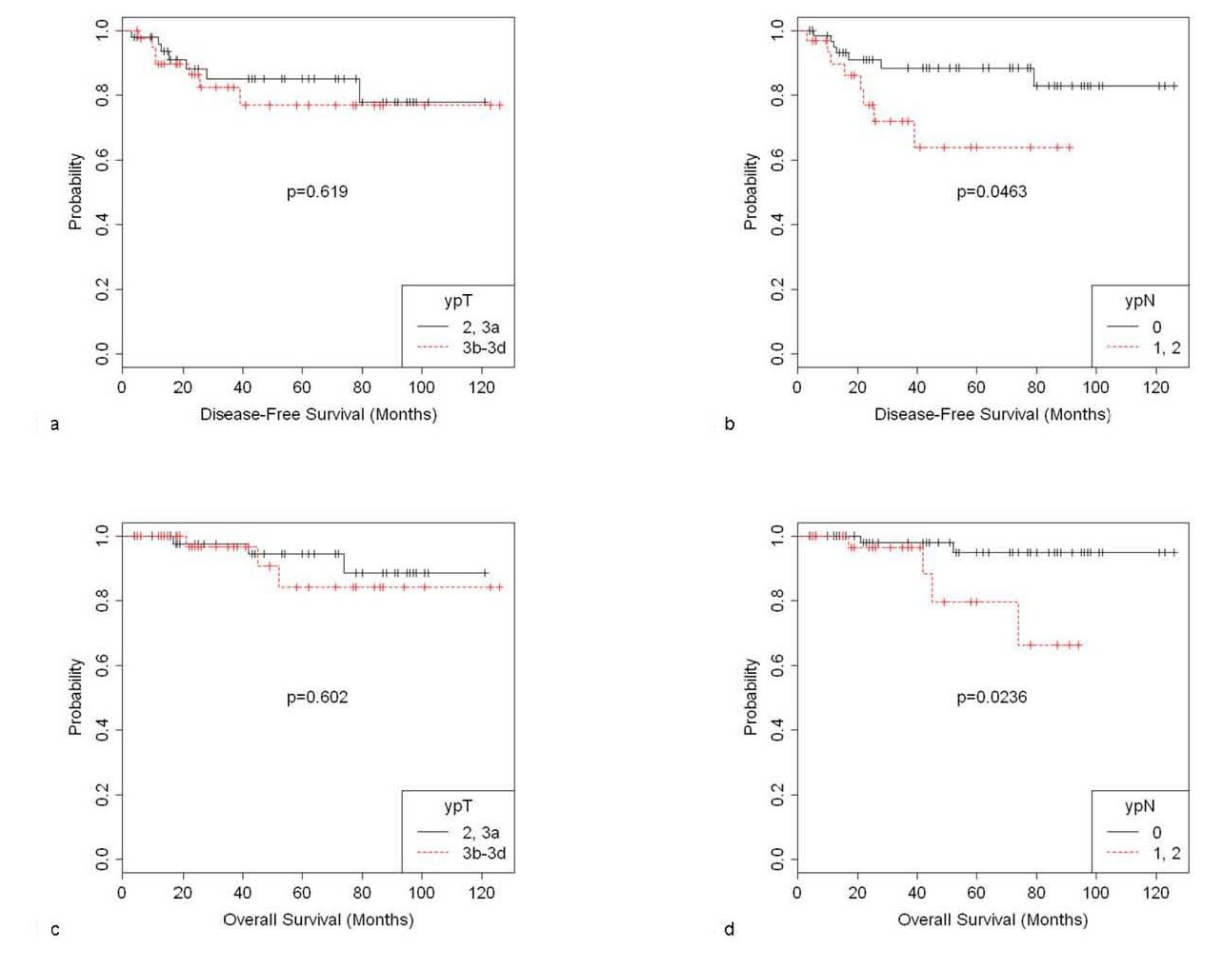
**DFS in patients with rectal cancer and intermediate response to preoperative RCT stratified by ypT stage (3a) and ypN stage (3b)**. OS in patients with rectal cancer and intermediate response to preoperative RCT stratified by ypT stage (3c) and ypN stage (3d).

Compared to the ypT2/3a stage, advanced residual infiltration into the mesorectal compartment (ypT3b-d) after preoperative RCT was not associated with a significantly decreased DFS (77% vs. 85%, p = 0.619) and OS (84% vs. 94%, p = 0.602) (Figure 3a and 3c). However, residual nodal involvement after preoperative RCT (ypN1/2) appeared as an important parameter for abbreviated DFS (88% vs. 64%, p = 0.0463) as well as OS (95% vs. 80%, p = 0.0236) (Figure 3b and 3d). In multivariate analyses, a persistent positive nodal status could be confirmed as an independent factor for poor DFS (p = 0.035). For OS, the significance failed however in the multivariate approach (p = 0.053) (Table 4).

**Table 4:** Comparison of DFS and OS with respect to ypT, ypN status and Tumor Grading

Parameter	Variable	Estimated 5 Year DFS Probability (%)	p-value: *univariate **multivariate	Estimated 5 Year OS Probability (%)	p-value: *univariate **multivariate
**ypT**	2/3a	85	*0.6	94	*0.6
				
	3b-d	77	**0.56	84	**0.7

**ypN**	0	88	*0.04	95	*0.02
				
	1/2	64	**0.03	80	**0.053

**Residual Tumor Differentiation**	High	88	*0.04	94	*0.09
				
	Low	62		71	

The probability of cancer relapse and distant metastases was stage-dependent. There was no significant difference within the group of nodal-negative patients with stage I and II disease (ypT2/3a N0 and ypT3b-d N0: 91% and 88%) or within the group of stage III patients (ypT2/3a N+ and ypT3a-d N+: 55% and 67%).

Residual mesorectal tumor infiltration (ypT3b-d) - though without immediate impact on survival - was significantly associated with occurrence of metastatic lymph node involvement after preoperative RCT (p < 0.001), which itself is an independent prognostic factor for survival.

Histologic tumor differentiation grading after RCT had a significant influence on DFS (p = 0.04), whereas patients with well and moderate tumor differentiation (high grade residual tumors) showed a tendency for prolonged OS (94% vs. 71%, p = 0.09). Furthermore, histologic tumor differentiation grading after RCT correlated with residual lymph node metastases (p < 0.001) as well as mesorectal tumor infiltration (ypT3b-d) (p = 0.0001).

### ypN0: Relevance of Pretherapeuthical Nodal Status?

When evaluating the 63 patients with ypN0 status for their pretherapeuthical nodal status (cN), staged by ErUS and MRI, 29% (n = 18) of patients had previous cN0 status and 71% (n = 45) presented with cN+ status. DFS and OS did not significantly differ in patients who initially presented with clinical evidence of mesorectal lymph node involvement but resulted in ypN0 after RCT (p = 0.46 and p = 0.54, respectively). Patients with clinically staged III rectal cancers therefore showed no higher risk of cancer relapse and cancer-related death than initially node-negative patients, as long as sterilization of lymph node metastases can be achieved with RCT.

## Discussion

Recent results from the randomized multicenter trial CAO/ARO/AIO-94 showed an enhanced local control and sphincter preservation with concurrently decreased toxicity after preoperative long-term RCT compared to postoperative RCT [6]. These results led to the recommendation of preoperative RCT in locally advanced (stage II/III) rectal cancers [7]. Preoperative RCT results in a very heterogeneous tumor response, which can be measured by various response parameters such as T-level downsizing, tumor downstaging, elimination of lymph node metastases, and pathomorphologic tumor regression.

Of 153 patients with stage II/III rectal cancer who received standardized preoperative RCT within randomized clinical trials, pCR as a major response criterion, was achieved in 16% (n = 10) of patients. pCR rates vary between 10 and 20% and were associated with a favorable outcome [8,10]. Nevertheless the majority of rectal cancers (70% of the actual collective) show intermediate response with residual tumor either within (ypT2) or beyond (ypT3) the rectal wall (Figure 2).

It remains unclear which subgroup of patients with intermediate response can be considered as cured after preoperative RCT and subsequent TME surgery. Conversly, it is of enormous clinical interest to know which subgroup necessitates adjuvant systemic therapy.

Involvement of circumferential resection margins (CRM) has recently been described as a very strong prognostic factor after preoperative short term radiation [22]. Although this is distinctly reasonable, fortunately only a considerable small group of patients is affected by positive CRM after preoperative long-term RCT. In our study, 7% of patients (n = 8) presented with cT4 status and potential CRM involvement in pretherapeuthical imaging. RCT-induced tumor downsizing was achieved in all cases, resulting in a maximal residual mesorectal infiltration of ≥ 1.5 cm (ypT3d) in 2 patients (2%). Pathologically confirmed complete (R0) resection with negative (>1 mm) CRM after RCT was accomplished in all patients including those previously classified as high-risk for positive CRM.

Prior to implementation of neoadjuvant strategies for rectal cancer, a tumor invasion of ≥ 5 mm into the mesorectal compartment, besides circumferential involvement, was described as a significant prognostic factor [23]. The decision to apply postoperative radiation or radiochemotherapy, was based on tumor invasion as well as a positive nodal status, and led to reduced recurrence rates and prolonged survival [24].

We therefore evaluated the impact of intramural depth of tumor invasion (ypT2) together with minimal (<1 mm) transgression of the muscularis propria (ypT3a) compared to a distinct transmural tumor invasion into the mesorectum (>1 mm; ypT3b-d). Since patients with ypT3a status show only an extremely marginal infiltration of the mesorectal compartment (<1 mm) we consider them to prognostically belong to the ypT2 group rather than to the tumors with distict mesorectal infiltration. Our results underline this assumption showing an increased incidence of nodal metastases in ypT3b-d patients compared to ypT2/3a patients.

In the patients presenting with previous cT3/4 rectal cancers (only 1 patient had cT2 N+ status, according stage III) the RCT-induced regression of tumor invasion depth to ypT2/3a status had no impact on prolonged DFS and OS. Thus, residual tumor transgression into the mesorectum after preoperative RCT showed no significant influence on cancer recurrence, providing that complete resection with negative CRM is achieved by adequate TME surgery.

Tumor downsizing from the extramural mesorectal compartment into the actual rectal wall therefore seems to be of importance only when tumor-free CRM and R0-resection cannot be guaranteed (former T3d/4 status).

In contrast to ypT, nodal status after preoperative CRT (ypN) significantly influenced cancer recurrence and overall survival in stage II/III rectal cancer patients with intermediate response within our investigation. This finding coincides with previous results and supports recent investigations with considerable numbers of patients [25,26] but it is based on a collective of patients with highly standardized diagnostic and treatment procedures according to the protocols of the respective clinical phase II and III trials of the German Rectal Cancer Study Group.

In agreement with other authors [25,27], we observed that pretherapeutical nodal involvement (cN+) has no impact on the prognosis of patients, in which ypN0 status can be achieved. Patients with evidence of lymph node involvement in pretreatment staging can therefore not categorically be considered as high risk for cancer relapse.

Anyway, patients with ypN+ status should be considered for upcoming trials with intensified adjuvant CT regimes as this might be more efficient in preventing systemic tumor relapse. Nonetheless, mesorectal tumor invasion (ypT3b-d) was significantly associated with residual lymph node metastases after RCT in our study (p < 0.001). We interpret this finding with a generally lower response to RCT regarding both downsizing of the primary tumor and sterilization of lymph node metastases. This might be due to improved biological behavior and enhanced resistance to RCT in individual cancers. The prognostic impact of mesorectal tumor infiltration remains unclear. We could not show straight effects on tumor recurrence and survival but are well aware that this might be due to the relative small number of patients underlying this investigation.

Neoadjuvant RCT has repeatedly been accused of reducing lymph node yield in rectal cancer specimens [28-31]. It has also been reported that the number of detected nodes in stage II rectal cancer patients influences survival [32-34]. Within our investigation, we evaluated a median number of 21 lymph nodes per specimen. In contrast to other investigations [35], we found no significant difference in lymph node yield between ypN0 patients with and those without subsequent development of distant metastases and tumor-related death. This might be explained by the implementation of extensive lymph node recovery at our institution and a minor variance of evaluated lymph node numbers between both groups.

While histologic tumor grading in colorectal cancers after primary surgery has been ascertained as a prognostic factor [13], its prognostic relevance following preoperative RCT remains unclear and currently does not belong to standard pathologic staging in rectal cancer specimens. Our results show that histologic grading of residual tumor cells is a reliable parameter, which correlates with advanced tumor biology and has straight impact on DFS despite RCT-induced histomorphologic alterations of the tumor. Thus, histopathologiclogic grading of residual tumor cells should be considered within risk stratification in rectal cancers after RCT.

Not unexpectedly, lymph node status displays as the major criterion for therapy stratification after application of preoperative RCT within our study and several recent investigations and might subdivide patients with need of intensified adjuvant treatment from those who can be considered as cured after surgery. In contrast Collette et al. [15], who reported the results of the EORTC 22921 trial, underlined that only patients with RCT-induced tumor downsizing to ypT1/2N0 status benefited from adjuvant CT. They interpret their results with an increased sensitivity to preoperative RCT as well as postoperative CT in this subgroup. However, 5-FU monotherapy was used in both, neoadjuvant and adjuvant setting in this trial. This might explain the failure of adjuvant CT in patients with a minor response to preoperative RCT. Thus, in the adjuvant setting an intensified or combined CT should be applied with different anti-tumoral mechanisms (e.g. FOLFOX/FOLFIRI regime ± targeted therapy) in patients with minor response to neoadjuvant treatment.

To date, most patients with positive nodal status after preoperative RCT will intuitively get adjuvant CT. Prospective randomized clinical trials should therefore clarify the impact of adjuvant treatment in patients undergoing preoperative RCT and radical surgery. For ypN0 patients 5-FU based adjuvant CT was shown as a potential overtreatment and had no significant effect on survival [36,37].

Nevertheless, in our actual study population, 7 patients with ypN0 status developed distant metastases during follow-up. All 7 had poorly differentiated residual tumors (low grade). Poor differentiation of residual tumor cell clusters after RCT and advanced invasion depth turned out to be predictors of lymph node metastases and may be indicators of occult nodal (micro-) metastases in patients classified as ypN0. Both parameters should thus be taken into account in ypN0 patients, particularly in cases of minor lymph node recovery, and might have influence on the decision for adjuvant CT.

Although this investigation is based on a homogeneous collective of patients treated within randomized clinical trials with replicable and standardized diagnostic and therapeutic procedures, its principal limitations are the retrospective character and the relatively small number of patients. Thus this study does not want to claim to ultimately answer the question which subgroup of patients need adjuvant CT after preoperative multimodal treatment and subsequent R0-resection. Prospective randomized trials will have to clarify the debatable role of postoperative CT in rectal cancer patients after preoperative RCT and radical TME surgery. The clinicopathologic parameters investigated in this study might give indications to stratify patient groups with lower and higher individual risk of tumor relapse and tumor-related death within future clinical trials.

## Competing interests

The authors declare that they have no competing interests.

## Authors' contributions

TS prepared the study design, assembled and analysed the data and drafted the manuscript. HR carried out the pathological diagnostics of the rectal cancer specimens and reviewed the manuscript. KJ carried out the statistical analyses. HC contributed the radiation therapy data and reviewed the manuscript. LC participated in assembling of the data and reviewed the manuscript. BMG and HB reviewed the manuscript. TL supervised the study and data assembling and critically reviewed the manuscript. All authors read and approved the final manuscript.
